# Rare Breast Carcinoma with Paradoxical Plasma Cell Immunoprofile: A Case Report

**DOI:** 10.3390/medicina56020062

**Published:** 2020-02-05

**Authors:** Sebastian Grădinaru, Mihai-Ciprian Stoicea, Liliana Mocanu, Iulian Antoniac, Daniela Gheorghiță, Alina Gabriela Mihaela Grigore

**Affiliations:** 1 IV Surgical Department, University Emergency Hospital, 050098 Bucharest, Romania; gradinarusebastian@gmail.com; 2 Department of Surgery, Faculty of Medicine, “Carol Davila” University of Medicine and Pharmacy, 050474 Bucharest, Romania; 3 Department of Pathology, Regina Maria Central Laboratory, 060044 Bucharest, Romania; mihai.stoicea@reginamaria.ro (M.-C.S.); georgescu.alina@gmail.com (A.G.M.G.); 4 Department of Pathology, County Emergency Hospital, 900591 Constanța, Romania; ilianamcn@gmail.com; 5 Faculty of Materials Science and Engineering, Politehnica University of Bucharest, 060042 Bucharest, Romania; antoniac.iulian@gmail.com

**Keywords:** lobular carcinoma, plasma cell differentiation, multiple myeloma

## Abstract

Plasma cell features are encountered in a variety of non-plasma cell neoplasias, especially carcinomas of a discohesive type, such as those occurring in the digestive tract and breast. Lobular carcinomas of the breast present themselves in a variety of architectural patterns and many cell morphologies, including plasmacytoid types. A matching plasma cell phenotype is sometimes an associated feature. We report a case of a moderate grade invasive lobular carcinoma with focal plasmacytoid morphology and aberrant expression of plasma cell markers in a patient previously diagnosed with multiple myeloma. Paradoxical plasma cell immunoprofiles can be encountered in many malignancies, causing serious diagnostic problems, even more so with those occurring in discohesive carcinomas in multiple myeloma patients.

## 1. Introduction

Case reports play an important role in clinical practice, as well as literature reviews, studies, and investigations, all of them being valuable resources [1,2,3,4,5,6,7,8,9]. Plasma cell neoplasias are currently diagnosed on histological specimens using morphological as well as immunophenotypical features, including expression of CD38, CD79a, MUM1, CD56, and CD138 (or syndecan), a transmembrane (type I) heparan sulfate proteoglycan with significant roles in epithelial organization [10], functioning like a receptor for extracellular matrix with roles in cell adhesion. CD138 is expressed in a variety of epithelial tumors, benign or malignant, such as keratoacanthoma and squamous cell carcinoma, but also in plasma cell myeloma, plasmablastic lymphoma, and primary effusion lymphoma. Epithelial tumors expressing CD138 do not usually express other plasma cell markers; in breast carcinomas particularly, CD138 is the most frequently encountered while CD56 is rarely seen [11].

Immunoglobulin light chains kappa and lambda show proportionate expression in non-neoplastic conditions, with restricted expression of either one in amyloidosis and various hematological neoplasias, including plasma cell myeloma [10]. While the expression of CD138 in epithelial tumors is well documented in literature, light chain expression is not. An associated expression of CD138 and light chains in an epithelial neoplasm poses a serious diagnostic challenge, especially in discohesive types of tumors including plasmacytoid phenotypes, such as those originating in the digestive tract, most often in the stomach, and invasive lobular carcinomas. In cases where a prior diagnosis of plasma cell myeloma exists, immunohistochemistry proves a reliable tool to separate carcinomas masquerading as plasma cell myeloma and vice-versa [12,13]. For such presentations, an extensive panel of antibodies should be employed in order to specifically characterize carcinomas, including various cytokeratins and less EMA (epitelial membrane antigen) (expressed by most plasma cell myelomas), and also hematological markers that should not be expressed (CD79a and MUM1).

We report a case of lobular carcinoma with a very unusual immunohistochemical profile, including extensive CD138 and light chain expression in a patient previously diagnosed with multiple myeloma, aiming to document this exceptional presentation.

## 2. Materials and Methods

A 66-year-old female patient diagnosed with multiple myeloma three years ago, subjected to chemotherapy and in remission at current presentation, reported a recent nodular growth in her right breast. The lesion was rendered suspect at imagistical evaluation and biopsied for histopathological examination. Following fixation for 24 h in 10% buffered formalin, the biopsy fragments were processed automatically and included in paraffin with strict thermal control (maximum 60 °C).

The paraffin block was sectioned in 2 µm thick sections, the first of which were stained conventionally (hematoxylin and eosin) on an automated stainer. Immunohistochemical testing was performed on a completely automated platform (Benchmark ULTRA, Hoffmann–La Roche, Basel, Switzerland), from deparaffinization to hematoxylin counterstaining. The evaluation itself included 19 antibodies marked CE-IVD (Table 1), with technical validation through internal and external quality assessment procedures. Conventional and immunohistochemical slides were evaluated by two pathologists independently with matching results and scores. The slides were scanned using an iScan Coreo Digital Scanner and referred for analysis to a third pathologist in the laboratory where the paraffin block originated, with congruent results.

## 3. Results

Clinical evaluation revealed a solid tumoral nodule imprecisely contoured, firm, 1.5 cm in greatest dimension, highly suspicious for malignancy. Clinical laboratory testing included blood counts and negative Bence Jones protein in urine. Ultrasonographic examination of the breast indicated a suspect lesion which was biopsied for histopathological examination.

**Figure 1:**
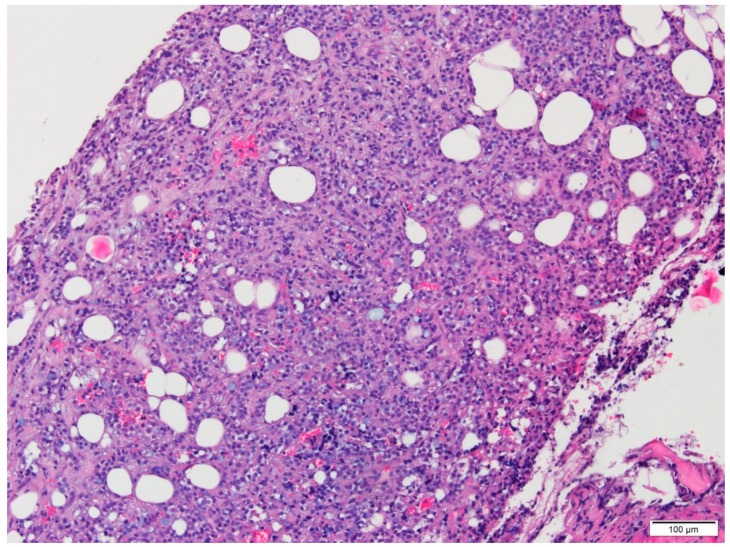
Conventional aspect of the lesion infiltrating adipose tissue (HE, 10×).

Microscopic examination of conventional stained slides revealed breast biopsy fragments with chronic inflammatory infiltrates, predominantly lymphocytic, with rare plasma cells, organized in groups around ductal–acinar structures, blood vessels and diffusely dispersed in the interstitium. One of the fragments presented massive tumoral infiltration (Figure 1), histopathologically consistent with invasive lobular carcinoma growing in solid nests and sheets with marginal single files of discohesive, equidistant cells, some of them with cytoplasmic microlumina, in a reduced sclerohyaline stroma with diffuse lymphocytic infiltrate. The tumoral infiltration was categorized as moderately differentiated (G2 histological grade) corresponding to a Nottingham score of 7: no duct formation (Df = 3 points), marked nuclear pleomorphism (A = 3 points) with occasional plasmacytoid marginal cells and rare mitotic figures (3/10 high power fields at 40×/0.45) (M = 1 point). Tumoral necrosis was not identified. No in situ component was present. Tumor emboli were not detected. Perineural invasion could not be determined because no resting nervous structures were identified.

**Figure 2:**
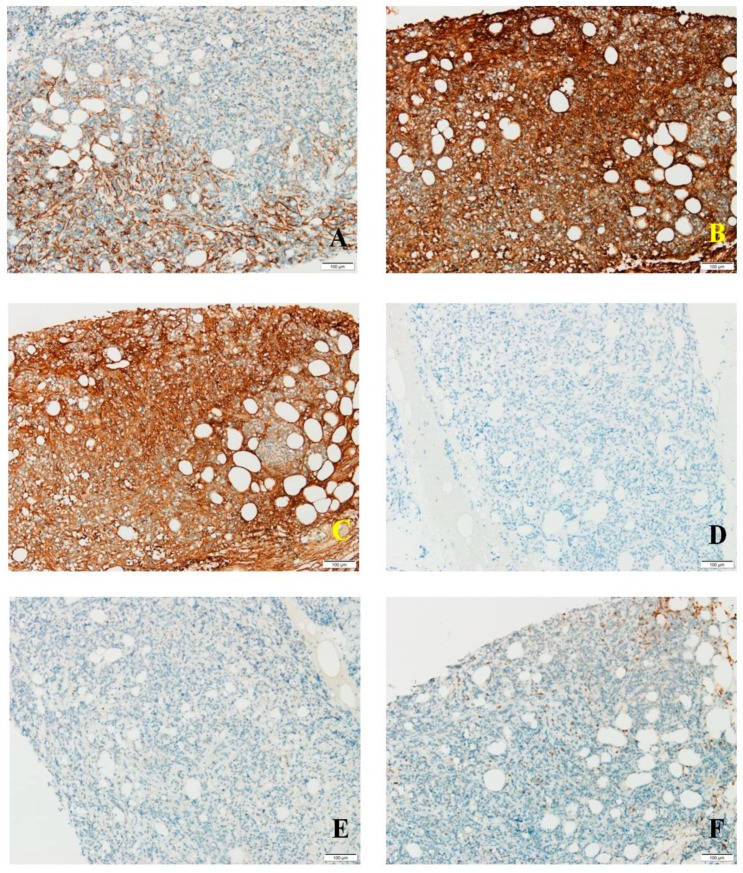
(**A**) CD138 staining in tumor cells; (**B**) kappa light chain expression in tumor cells; (**C**) lambda expression in tumor cells; (**D**) negative CD79a reaction in tumor cells; (**E**) MUM1 absent in tumor cells; (**F**) CD3 expression in peritumoral and rare intratumoral T cells.

Tumor cells expressed CD138 in a predominantly cytoplasmic manner, with incomplete membrane staining and focal granular reaction, in up to 25% of cells (Figure 2A). Aberrant kappa expression was noted in −70% of tumor cells with variable, predominantly high intensity (Figure 2B). Lambda was identified in 30% of tumor cells, unusual high-intensity expression (Figure 2C). No reaction recorded for CD79a and MUM1 in tumor cells (Figure 2D,E). CD20 was observed in relatively rare mature B lymphocytes dispersed interstitially, with no reaction in tumor cells. The peritumoral inflammatory infiltrate consisted mostly of T lymphocytes positive for CD3 (Figure 2F), most of them CD4 positive, with rare extratumoral CD8 positive cells and CD56 positive cells. Tumor cells were negative for CD56.

**Figure 3:**
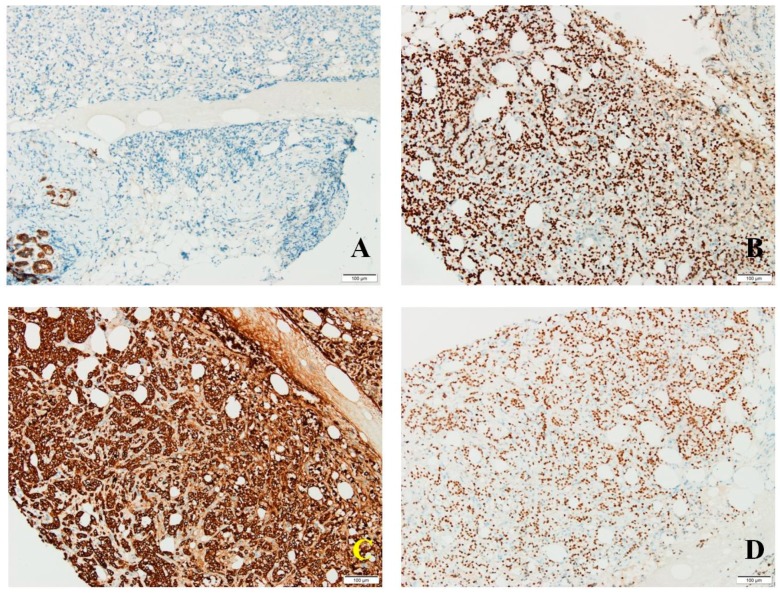
(**A**) No cadherin E expression in tumor cells; positive membrane reaction in resting ductal–acinar structures; (**B**) diffuse GATA3 expression in tumor cells; (**C**) intense diffuse CK8/18 expression in tumor cells; (**D**) estrogen receptor expression in frequent tumor cells.

Cadherin E was absent in tumor cells; positive membrane reaction was observed in resting ductal–acinar structures (Figure 3A). GCDFP15 was distributed in a zonal fashion in tumor areas and resting ductal–acinar structures, while GATA3 (transcription factor encoded by GATA3 gene) showed intense diffuse nuclear positivity in tumor cells (Figure 3B). CK8/18 positivity matched the diffuse GATA3 pattern (Figure 3C), thus excluding a plasma cell myeloma.

Estrogen receptors were intensely expressed in 90% of tumor cells (Allred score 8, H score 270) (Figure 3D); progesterone receptors were seen in 15% of tumor cells (Allred score 5, H score 23); androgen receptors presented a predominantly moderate expression in 90% of tumor cells (Allred score 7, H score 150). Her2 scored 0 with no membrane staining in tumor cells. Proliferation index Ki67 was expressed in 30% of tumor cells, thus suggesting a Luminal B molecular subtype.

The case was classified as invasive lobular carcinoma with paradoxical plasma cell differentiation including aberrant expression of CD138 and light chains (kappa/lambda ratio 2.33:1), Luminal B subtype.

The patient was referred for further clinical and imagistical evaluation for staging purposes. No supplementary suspect lesions were present at imagistical evaluation. Upon the publication of this material, no futher case details were available.

## 4. Discussion

Lobular carcinoma of the breast can present in various architectural patterns including solid nests and sheets. Also, cell morphology can be diverse, from monomorphous appearing cells with little cytoplasm, to plasmacytoid cells, signet ring cells, and pleomorphic nucleated cells [14,15]. The particular plasmacytoid morphotype can cause problems in patients with a history of plasma cell myeloma, such as the one reported by us, even if the lesion is located in the breast or bone, marrow or any other site—reports of such confusing lesions with aberrant CD138 expression rely on detailed immunohistochemical analysis to elucidate the diagnosis [12,13]. Lobular carcinomas contain, most of the time, an admixture of cells including plasmacytoid cells and many times express CD138 not only at membrane level, but are also aberrantly cytoplasmic. This type of expression is also noted in cohesive types of breast carcinomas, in quite significant numbers (e.g., 18 of 26 in one series) [11]. Occasional stromal cell reactivity for CD138 is noted in breast carcinomas where tumor cells lack expression of this marker; there is no clear significance for this type of reactivity, nor any proven relationship between CD138 positive stromal cells and CD138 positive tumor cells [16]. Normal breast stroma or benign stromal or stromal–epithelial proliferations lack CD138 expression. In our case, aberrant cytoplasmic CD138 reaction was also noted in stromal cells.

CD138 is found in other types of carcinomas, most frequently in squamous cell carcinomas indifferent of histogenesis, and in more than half of basal cell carcinomas [11], ovarian carcinomas, endometrial carcinomas, liver and lung carcinomas, prostate carcinomas, thyroid and adrenal carcinomas [11,16,17]. CD138 expression is a very rare phenomenon in mesothelioma and melanoma, thus serving as a potential differential tool between these tumors and carcinomas [17].

Paradoxical expression of kappa and lambda light chains with a rather normal ratio (3:1 in serum, 2:1 in tissue) without restriction to one particular type should not pose particular problems since there is no clonality suggested in this case. It is rather unusual to see more than one plasma cell marker expressed in lobular carcinomas; however, lack of CD79a and MUM1 discard the plasma cell neoplasia scenario. Although plasma cell myeloma cells express EMA, cytokeratin is generally negative. In our case, CK8/18 high expression rules out a plasma cell proliferation in favour of breast carcinoma, doubled by the diffuse GATA3 expression, GCDFP15 expression, and hormonal receptor status. To the best of our knowledge, this is a unique report of breast carcinoma exhibiting multiple plasma cell features in a patient previously diagnosed with and treated for plasma cell neoplasia.

The prognostic impact of plasma cell immune profile in breast carcinomas is unknown. There are studies suggesting that CD138 loss in certain types of carcinomas, especially squamous cell carcinoma of the head and neck region, signals a potential aggressive course of the disease due to loss of adhesion and, in most cases, an epithelial–mesenchymal transition in place, while preserved expression foresees a favorable outcome; similar suggestions have been made for carcinoma cells in effusions, but this needs further investigation to clarify [14]. Where immunoglobulin light chain expression stands, there is no definite prognostic impact quantified in breast carcinomas. Most probably the quintessential morphological and immunophenotypical prognostic information resides in the discohesive character and molecular subtype of the tumor cells.

Upon the publication of this case report, the biological and clinical status of the patient indicated no sign of recurring plasma cell myeloma by clinical laboratory investigations and imagistics. Correlated with the immunohistochemical data concerning tumoral cells and adjacent stromal cells and inflammatory cells, a coexisting plasma cell myeloma and invasive lobular carcinoma scenario could be irrevocably ruled out. The patient is currently monitored similar to any other case of metachronous neoplasias.

## 5. Conclusions

Plasma cell marker expression in breast carcinomas is a paradoxical phenomenon that can cause diagnostic dillemas. It is advisable not to use plasma cell markers routinely in breast carcinomas since aberrant expression might be encountered in significant numbers, especially CD138. However, the use of these antibodies cannot be avoided in cases with preexisting or concurrent plasma cell neoplasia. In such presentations, a fairly extensive immunohistochemical panel and correlation with imagistic and biological data ensure a clear distinction between carcinoma and plasma cell myeloma.

## Figures and Tables

**Table 1:** Immunohistochemical antibodies employed for the case.

Antibodies Employed	Kit, Producer	Clone
CD79a	CONFIRM anti-CD79a Rabbit Monoclonal Primary Antibody, Ventana	SP18
CD138	CD138/syndecan-1 Mouse Monoclonal Antibody, Cell Marque	B-A38
MUM1	Rabbit Monoclonal Antibody, Cell Marque	MRQ-43
E-CD	E-Cadherin, Cell Marque	EP700Y
GCDFP-15	GCDFP-15, Cell Marque	EP1582Y
GATA3	Mouse Monoclonal Primary Antibody, Cell Marque	L50-823
K (Kappa)	CONFIRM anti-Kappa Rabbit Polyclonal Primary Antibody, Ventana	polyclonal
λ (LAMBDA)	CONFIRM anti-Lambda Rabbit Polyclonal Primary Antibody, Ventana	polyclonal
CK8/18	Cytokeratin 8 and18, Cell Marque	B22.1 & B23.1
Ki67	CONFIRM™ anti-Ki-67 Rabbit Monoclonal Primary Antibody, Ventana	30-9
ER	CONFIRM™ anti-Estrogen Receptor Rabbit Monoclonal Primary Antibody, Ventana	SP1
PR	CONFIRM™ anti-Progesterone Receptor Rabbit Monoclonal Primary Antibody, Ventana	1E2
Her2	CONFIRM anti-HER-2/neu Primary Antibody, Ventana	4B5
AR	anti-Androgen Receptor Rabbit Monoclonal Primary Antibody, Cell Marque	SP107
CD3	CONFIRM anti-CD3 Rabbit Monoclonal Primary Antibody, Ventana	2GV6
CD20	CONFIRM anti-CD20 Monoclonal Primary Antibody, Ventana	L26
CD4	CONFIRM anti-CD4 Rabbit Monoclonal Primary Antibody, Ventana	SP35
CD8	CONFIRM anti-CD8 Rabbit Monoclonal Primary Antibody, Ventana	SP57
CD56	CD56 Rabbit Monoclonal Antibody, Ventana	MRQ-42
